# Normothermic frozen elephant trunk without circulatory arrest

**DOI:** 10.1007/s12055-023-01536-1

**Published:** 2023-06-14

**Authors:** Paolo Berretta, Michele Galeazzi, Pietro Giorgio Malvindi, Olimpia Bifulco, Beatrice Buratto, Mariano Cefarelli, Jacopo Alfonsi, Filippo Capestro, Emanuele Gatta, Marco Di Eusanio

**Affiliations:** 1 Polytechnic University of Marche, Ospedali Riuniti, Ancona, Italy; 2 Cardiac Surgery Unit, Lancisi Cardiovascular Center, Ancona, Italy; 3 Cardiac Surgery Unit, Department of Cardiac, Thoracic, Vascular Sciences and Public Health, University of Padua, Padua, Italy; 4 Vascular Surgery Unit, Aortic Team, Lancisi Cardiovascular Center, Ancona, Italy

**Keywords:** Aorta, Aortic surgery, Aortic arch

## Abstract

We describe our technique for total aortic arch replacement with stenting of the descending thoracic aorta allowing normothermic cardiopulmonary bypass and avoiding hypothermic circulatory arrest.

**Supplementary information:**

The online version contains supplementary material available at 10.1007/s12055-023-01536-1.

In the last years, aortic arch surgery with frozen elephant trunk (FET) technique has been progressively showing improved clinical outcomes, due to refined surgical techniques and organ protection methods. Nonetheless, long cardiopulmonary bypass (CPB) and hypothermic circulatory arrest (HCA) times still impact negatively on patients’ outcome. Hereby, we present a FET technique that allows normothermic CPB and avoids HCA [1]. The main features are (1) uninterrupted total body perfusion by femoral artery and brachiocephalic trunk (BCT) cannulation; (2) complete antegrade selective cerebral perfusion (ASCP); (3) retrograde endograft delivery through femoral access; and (4) aortic endoclamping [2].

After full sternotomy, the aorta and the arch vessels are carefully prepared. Both the BCT and the femoral artery are cannulated. The retrograde deployment of an endograft is completed through contralateral femoral access. Normothermic CPB is started; the left subclavian artery is transected and cannulated for ASCP through interposition of an 8-mm graft. After cardioplegic arrest, ASCP is completed by cannulation of the left carotid artery. The BCT is occluded proximally and a balloon catheter is used to occlude the endograft; this allows continuous lower body perfusion from the femoral artery inflow line, while ASCP keeps the brain fully perfused. Distal anastomosis between a Terumo Siena 4-branched vascular graft and the endograft is performed. The Siena graft is clamped, the balloon deflated, and the proximal anastomosis completed. Arch vessels are then reimplanted in the standard fashion.

We do believe that by the complete avoidance of HCA, our normothermic FET technique may improve clinical results after FET. Currently, we successfully performed 19 procedures in selected patients presenting with degenerative aneurysm (*n* = 17) and aortic dissection (*n* = 2). It has to be noted however that patients with aortic dissection are not the most suitable cases to be treated with this technique due to the risk of aortic injury following endoclamping or inadequate endograft sealing.

### Supplementary Information

Below is the link to the electronic supplementary material.

Supplementary file1 (MP4 348982 KB)

## Data Availability

The data that support the findings of this study are available on request from the corresponding author. The data are not publicly available due to privacy or ethical restrictions.
